# Isolation and characterization of bacterial cellulose produced from soybean whey and soybean hydrolyzate

**DOI:** 10.1038/s41598-023-42304-w

**Published:** 2023-09-25

**Authors:** Xin Liu, Liang Cao, Shenao Wang, Li Huang, Yu Zhang, Miaoyi Tian, Xuejiao Li, Jinyou Zhang

**Affiliations:** 1 Department of and Chemical and Pharmaceutical Engineering, School of Materials Science and Chemical Engineering, Harbin University of Science and Technology, Harbin, 150040 China; 2 Heilongjiang Bayi Agricultural University, Daqing, 163319 China

**Keywords:** Biotechnology, Materials science

## Abstract

Soybean whey and soybean hydrolyzate can be used for the biotechnological production of high-value products. Herein, we isolate soybean whey (SW)-and soybean hydrolyzate (SH)-derived bacterial cellulose (BC, produced by kombucha) and characterize it by a range of instrumental techniques to reveal differences in micromorphology, crystallinity, and themal behavior. Studies have shown that the amounts of wet state BC produced from HS, SW and SH was 181 g/L, 47 g/L and 83 g/L, respectively. The instrumental analysis of BC, included SEM, AFM, FT-IR, XRD and TGA. It is shown that the FT-IR spectra of BC have a similar character, but we found differences in the micromorphology,crystallinity and thermal temperature of BC. The minimum average widths of the fibers produced from SH medium was 100 ± 29 nm. The CrI values of BC produced from SH medium was 61.8%. The maximum thermal degradation rate temperature of BC produced from SW medium was 355.73 °C. The combined results demonstrate that soybean industrial waste can be used as a cost-effective raw material for BC production.

## Introduction

Cellulose, an important biodegradable carbohydrate polymer comprising linear chains of β(1 → 4)-linked D-glucose units^1^, is the most widely distributed and most naturally abundant polysaccharide, accounting for more than half of the carbon content of the plant kingdom. Natural cellulose is usually found in combination with hemicellulose, pectin, and lignin, and is therefore subjected to purification, which leads to irreversible structural damage and environmental pollution^2^. Cellulose is mainly produced by vascular plants (such as trees and cotton) and some bacteria,e.g., *Acetobactor, Rhizobium, Pseudomounas, Sarcsna, Ahromobaerer, Aerobaerer, Alcalligenes, Azorobaerer*, and *Grobaererium*^3–5^. The morphology, structure, properties, and applications of bacterial cellulose (BC) are significantly dependent on bacterium type. Among the aforementioned bacteria, *Acetobacter glucobacteria* is currently the most widely studied strain that can be applied to a broad range of raw materials, features high production capacity, and is commonly used in scientific research and industrial production. The above bacterial strains are prone to contamination by other microorganisms, as cellulose production is an aerobic process^6^. BC has a molecular structure identical to that of plant cellulose and features the advantages of high purity, three-dimensional nanostructure, high crystallinity, biodegradability, hydrophilicity, and excellent mechanical properties resulting from the combination of an ultra-fine compact fiber network structure and high tensile strength^7,8^. Therefore, BC is considered to be an attractive biomaterial with potential for widespread applications in the paper, acoustic membrane, and pharmaceutical industries^9^. Moreover, BC is widely used in the food, paper, and the cosmetics industries, e.g., thick sheets of BC cooked in sugar syrup are used to supplement desserts, fruit cocktails, and jellies^10,11^, while BC aqueous suspensions are widely used to improve the rheology of processed foods^11,12^. Additionally, BC finds applications as a food contact packaging material, thickener, emulsifier, binder, stabilizer, and food ingredient. In the paper industry, BC is employed to increase the strength, water absorption ability, and durability of paper, and is also used in medicine as a component of wound dressings, artificial skin, and artificial blood vessels^13^.

BC surface morphology and physicochemical/ thermodynamic properties depend on the employed strain and culture conditions. Therefore, The research of bacterial cellulose focuses on strains, culturing conditions, which determines the focus of related research^14,15^.Despite the advantages of BC over plant cellulose, the production of the former is expensive and consequently not widespread, which has inspired research on the use of industrial and agricultural waste (e.g., waste beer yeast, acidic by-products of alcohol production, whey and sucrose molasses, potato peel waste acid hydrolysate, and coconut water) as a raw material to increase the yield and reduce the expense of BC production. Soy whey, is a by-product of soybean protein isolate production, is rich in sugar and protein, while soy hydrolyzate, a liquid by-product remaining after soybean powder is hydrolyzed by alkaline proteases to remove oil and protein is rich in nutrients such as small-molecule sugars, small-molecule proteins, and peptides. Therefore, these materials are considered to be promising substrates for the fermentation industry. For example, when soy whey was used as a medium for BC production, the yield of BC reached 4.10 g/L on the seventh day of cultivation under static conditions^16^.

In this study, we characterize BC produced from soybean whey and soy hydrolyzate without any extra supplementation, and investigate the effect of medium type on BC morphology, and thermal, chemical, and physical properties, proposing a waste-free and green way of BC production.

## Experimental

### Materials

*Kombucha* was purchased from the China Center of Industrial Culture Collection (Beijing, China). DongNong No. 42 (DN42) soybean samples were purchased from the Northeast Agricultural University Soybean Research Institute (Harbin, Heilongjiang Province, China). Puffed soybeans were provided by Jiu San Group Tieling Soybean Technology Co., Ltd. (Tieling City, Liaoning Province, China). Alcalase 6.0 L alkaline protease was purchased from Novozymes Biotechnology Co., Ltd. (Beijing, China). Peptone, and yeast extract were purchased from Beijing Aoboxing Biotechnology Co., Ltd. (Beijing, China). Glucose, Na_2_HPO_4_·12H_2_O, and citric acid were purchased from Tianjin Tianda Chemical Reagent Factory (Tianjin, China). Other chemicals were of analytical grade.

### Methods

#### Statement

We declare that the soybean samples (DN42) used in this experiment comply with the provisions of the Plant Management Law of the Government of China. The soybean samples (DN42) used in this experiment also comply with the guidelines of IUCN Species Survival Commission.

#### Preparation of SW and SH media

DongNong No. 42 soybean samples were stored at 4 °C in a cold room. Moldy and damaged seeds were removed, and the remaining seeds were washed and soaked in water for 6 h at room temperature. The soaked soybeans were drained, rinsed, and ground in a Joyoung soy milk maker (JYD-P11S81, Joyoung Co., Ltd., China).using a 1:10 (w/w) dry soybeans:water ratio. The obtained slurry was filtered through a 200-mesh sieve, and the isolated soymilk was boiled for 10 min, quickly cooled to room temperature, adjusted to pH 4.5 with 0.1 M acetic acid, and centrifuged at 8000 g for 15 min to remove deposits and impurities. The supernatant was adjusted to pH 6.0 with 0.1 M NaOH. to afford SW. SH was prepared using the method of Li et al.^17^. In particular, hydrolysis of puffed soy flour was performed with Alcalase 6.0 L (1.85%, v/w), and the reaction was maintained at pH 8.5, and 55 °C for 3 h.The mixture was centrifuged at 8000 g for 15 min,and the supernatant was adjusted to pH 4.5 with 0.1 M acetic acid, heated for 10 min, and centrifuged again at 8000 g for 15 min. Finally, the resulting supernatant was adjusted to pH 6.0 with 0.1 M NaOH to afford SH.

#### Bacterial strain and culturing conditions

In addition to SW and SH media, the Hestrin and Schramm (HS) medium, comprising glucose (20 g/L), peptone (5 g/L), yeast extract (5 g/L), citric acid (1.15 g/L), and NaHPO_4_ (2.7 g/L), was used for BC production^18^. All media had pH 6.0 and were autoclaved for 20 min at 120 °C.

To prepare the seed medium, *kombucha* from an agar plate was aseptically transferred into a 250-mL Erlenmeyer flask containing 100 mL of the medium. The seed medium was inoculated at 6 vol% and incubated at 28 °C for 24 h. BC was produced in 250-mL Erlenmeyer flasks containing 100 mL of HS standard medium, SW medium, or SH medium at 28 °C for six days under static conditions. The experiments were conducted in triplicate, and the results were reported as averages of three measurements. The nutrient composition of the new medium were determined. The resuls indicated that the main nutrient composition of SW were determined to be total sugar 16.04 ± 1.04 g/L, protein content 0.86 ± 0.017 g/L, the nutrient composition of SH were total sugar 20.04 ± 1.15 g/L, protein content 1.05 ± 0.048 g/L, and pH range 4.5–5.0.

#### BC quantitation and purification

Following incubation, BC samples were collected, thoroughly washed with distilled water to remove medium components, treated with 1 M NaOH solution at 80 °C to eliminate bacterial cells, extensively rinsed with 1 M acetic acid and then with distilled water until the pH of the water washing became neutral. Wet-state weights (*W*_w_) were measured, and the purified BC pellicles were freeze-dried and weighed to determine dry-state weights (*W*_d_). The cellulose fiber content (*R*_cf_) of pellicles was determined as

1 $$R_{{{\text{cf}}}} = W_{{\text{d}}} /W_{{\text{w}}}$$

#### Fermentation kinetics

To identify the efficiency of substrate conversion, we probed fermentation kinetics during BC production and evaluated BC production efficiency after six days of cultivation. Substrate conversion, BC production rate, and BC production yield were calculated as follows^19^:

2 $$Substrate\;conversion \left(\%\right)=\frac{{S}_{i}-{S}_{f}}{{S}_{i}}\times 100,$$

3 $$\mathrm{BC\;production\;rate}\left(\mathrm{g}/\mathrm{Ld}\right)=\frac{{m}_{BC}}{v\times t},$$

4 $$\mathrm{BC\;production\;yield}\left(\mathrm{\%}\right)=\frac{{m}_{BC}/v}{{S}_{i}-{S}_{f}}\times 100,$$

where *S*_i_ is the initial concentration of substrate in the medium (g/L), *S*_f_ is the final concentration of substrate in the medium (g/L), *m*_BC_ is the mass of produced BC (g), *v* is the reaction volume (L), and *t* is the reaction time (days).

#### Scanning electron microscopy (SEM) imaging

SEM (SU8010, HITACHI, Japan) micrographs of BC samples (sputter-coated with Au to a thickness of ~ 5 nm) were recorded at an accelerating voltage of 5.0 kV. and used for fibril width measurements.

#### Atomic force microscopy (AFM) imaging

BC surface morphology was probed by tapping mode AFM (Bioscope Resolve, Bruker, Germany). For imaging, BC samples were air-dried on a glass slide to afford thin films. A silicon nitride cantilever with a nominal pyramidal tip radius of 5 nm was used. The scan rates equaled 1.0 Hz/s, and image resolution equaled 256 × 256 points.

#### Fourier transform infrared (FTIR) spectroscopy

Smart attenuated total reflectance Fourier transform infrared (ITR-FTIR) spectroscopy (Nicolet iS10, Thermo Fisher, USA) in transmittance mode was used to determine structural differences between freeze-dried BC samples. For each sample, 16 scans at a resolution of 4 cm^−1^ were collected within a wave number range of 4000–500 cm^−1^.

#### X-ray diffraction (XRD) analysis

BC crystallinity was probed by XRD (D8 ADVANCE, Bruker AXS, Germany) measurements, which were carried out using Cu *K*_α_ radiation (*λ* = 0.15418 nm, 40 kV, 40 mA) at ambient temperature in the 2*θ* range of 5–50° at a step size of 0.02° and a scan speed of 1.5°min^−1^. The crystallinity index (CrI) was calculated as

5 $$\mathrm{\%CrI}=\frac{{I}_{100}-{I}_{am}}{{I}_{100}}\times 100,$$

where I_100_ is the peak intensity of the [1 0 0] reflection at 2θ = 14°, and I_am_ is the peak intensity of amorphous cellulose at 2θ = 18°.

The crystallite size (CrS) was estimated using the Scherrer quation:

6 $$\mathrm{CrS}\left(\mathrm{nm}\right)=\frac{k\uplambda }{\beta \mathrm{cos}\theta },$$

where k = 0.9 is the shape factor, λ = 0.154 nm is the X-ray wavelength, β is the full-width at half-maximum intensity in radians, and θ is the Bragg angle.

#### Thermogravimetric analysis (TGA)

The thermal stability of BC samples was probed by TGA in Oxygen atmosphere (Pyris 6 TGA, Perkin Elmer Co., Ltd., USA) at a scan rate of 10 °C min^−1^.

#### Statistical analysis

All experiments were performed in triplicate, and the results were expressed as means ± standard deviation. Analysis of variance (ANOVA) was performed using SPSS software (Version 25; SPSS Inc., USA). Duncan’s multiple-range testes were used to compare the differences between means at a 95% confidence level.

## Results and Discussions

### BC yield and production efficiency

**Figure 1:**
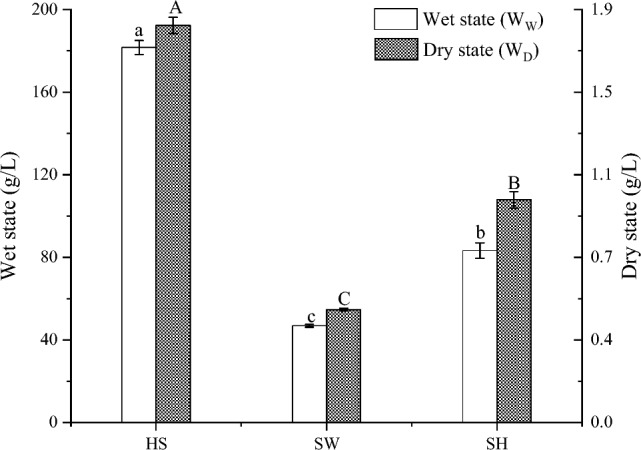
Amounts of BC produced by *Gluconacetobacter sp* in static culture conditions using standard HS medium, SW medium and SH medium for 6 days.

Figure 1 shows the amounts of BC produced by *Gluconacetobacter sp* under static culturing conditions for standard HS, SW and SH media over six days, According to these results, standard HS medium showed the highest BC yield compare with SW medium and SH medium. The W_W_ of standard HS medium was 182 g/L, The W_W_ of SW medium and SH medium were 47 g/L and 83 g/L, respectively. The W_D_ of standard HS medium, SW medium and SH medium were 1.78 g/L, 0.51 g/L and 1.00 g/L, respectively. It was confirmed that the composition of medium can influence BC yield. Although the yield of BC produced from soy by-product medium is lower than that of the standard HS medium. It is indicated that soy processing by-products can be used as a medium to synthesize bacterial cellulose. Later, we can improve the yield of bacterial cellulose by adding nutrients to the culture medium to optimize culture conditions. On the basis of the W_W_ and W_D_ values, the ration of cellulose fibers ${R}_{cf}$ was calculated (Table 1). The BC produced from HS showed the smallest ${R}_{cf}$ value. It Indicated that BC produced from standard HS medium has the highest water content.

**Table 1:** R_cf_ values, Kinetic studies parameters, CrI and CrS of BC produced from SH medium, SW medium and standard HS medium.

Medium	*R*_*cf*_	Substrate conversion ration (%)	BC production rate (g/Ld)	BC production yield (%)	CrI (%)	CrS (nm)
HS	0.0098	34.77	0.30	12.24	66.3	4.19
SW	0.0108	32.96	0.08	5.72	46.2	3.72
SH	0.0120	18.44	0.17	5.55	61.8	3.48

Kinetic parameters are not only important for estimating the bioprocess substrate consumption, but additionally to develop control strategies. without any supplementation of SH medium and SW medium to analyze in more details the production of BC. The results obtained are compared with those obtained using the standard standard HS medium. As shows in Table 1. The substrate conversion ration, BC production rate, BC production yield obtained for standard HS medium was higher than those of the SW medium and SH medium. It showed that the standard HS medium was more suitable for the production of BC. In comparison, a considerably low substrate conversion ration and BC production yield were obtained for SH medium, However, BC production rate was relatively high. We find that substrate conversion ratio and BC production yield have a certain correlation. Based on kinetic parameters, SH medium and SW medium can be used to produce BC. Therefore, determining the optimal nitrogen source and carbon source for BC production is the next step in the work.

### Surface morphology of BC

**Figure 2:**
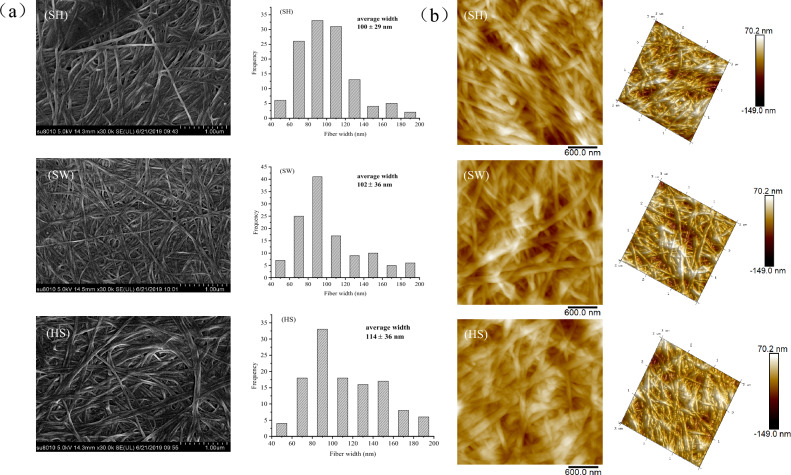
SEM (**a**) and AFM (**b**) images of BC microfibrils and distribution of width for fibrils (n = 100) produced from SH medium, SW medium, standard HS medium. The average widths were calculated from 100 randomly selected points on the fibers in SEM images.

Figure 2a shows the surface morphology of the freeze-dried BC observed by SEM. All BC samples show a structure consisting of rod-shaped nanofibers, which form a porous three-dimensional network structure. However, the fibers produced from standard HS medium look slightly thick and those of medium look slightly thin. The fibers produced from SW medium look slightly dense. It indicated that the medium component affects the morphological properties of the fibers. In fact, the distribution of widths of the fibers was analyzed and determined to be in the ranges 40–200 nm for the fibers. The cellulose width distribution in this study is similar to the previous report^20^. The average widths of the fibers produced from SH medium, SW medium and standard HS medium were 100 ± 29 nm, 102 ± 36 nm and 114 ± 36 nm, respectively. The widths of most of the fibers showed a wide distribution from 80 to 100 nm, and the distribution frequency of the fibers produced from SW medium was larger. The process of synthesizing bacterial cellulose can be divided into four processes of polymerization, secretion, assembly and crystallization. The synthesis of BC fibers is closely related to cellulose synthase, which exists in the cell membrane of bacterial^21^. First, cellulose synthase promotes the synthesis of 1.5 nm wide subfibers. second, the subfibers aggregate to form microfibrils, and then the microfibrils self-assemble into fibers. Therefore, the width of the fiber depends on the amount of cellulose synthase. The fibers produced from SH medium and SW medium in this study were slightly smaller than standard HS medium, suggesting that the number of cellulose synthase in standard HS medium may be larger. It has been known that the widths of the BC fiber depend on the kinds of bacterial, medium composition and culture conditions^20^.

To study the micro-morphology and microscopic details of its surface of BC, atomic force microscopy (AFM) was used. Figure 2b shows the surface morphology of the freeze-dried BC. The dense and aggregated typical BC structure is more apparent by AFM microscopy. Besides, agglomeration of BC microfibrils is also a result of the freeze-dried process. The morphology of BC samples displayed nano-scale network structure. Bacterial cellulose fibers were densely packed, irregularly ordered and rough. The surfaces of oven dried BC films were constituted of numerous randomly oriented and overlapped fibrils producing an aggregated web structure^22^. The samples produced from SH medium, SW medium and the standard HS medium were compared, There are differences in the width of the microfibrils. The BC microfibrils formed on standard HS medium was thicker. Similar observations, as in SEM images were observed in the width of cellulose microfibrils. The morphological changes of BC can influence various properties.

### Structural analysis of BC

**Figure 3:**
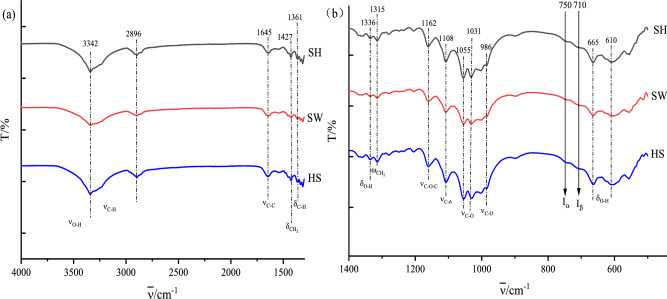
Comparative FTIR spectra of (**a**) 4000–1300 cm^−1^ and fingerprint region (**b**) 1400–450 cm^−1^of BC microfibrils produced from SH medium, SW medium, standard HS medium.

FTIR considered as an important characteristic tool for macromolecule as cellulose. To analyze the chemical structure of BC, FTIR measurements were carried out on the freeze-dried BC. For a more comprehensive and qualitative analysis and investigation of the FT-IR spectra, the FT-IR spectra were divided in two regions, 4000–1330 cm^−1^ and 1330–500 cm^−1^ (Fig. 3a,b). The high frequency region with a wave number between 4000 and 1330 cm^−1^ is called a feature region, and the low frequency region between 1330 and 500 cm^−1^ is called a fingerprint region. The spectrum of BC shows a high degree of similarity. The functional groups of BC samples obtained from fermentation using industrial waste liquid and standard HS medium were almost the same. The intensity of peak in fingerprint region of BC produced from SW medium is weaker than the other mediums. BC has many characteristic peaks refer to its function group^23^. The characteristic peaks of BC appeared at 3342 cm^−1^ for O–H stretching vibrations at -CH_2_OH groups. The broader peak for BC indicated stronger OH bonding. 2896 cm^−1^ for C-H streching vibrations at C-6. 1645 cm^−1^ for C-O streching vibrations for the glucose carbonyl group. 1427 cm^−1^ for CH_2_ symmetric bending, 1361 cm^−1^ for CH symmetric bending. 1336 cm^−1^ for OH in plane bending, 1315 cm^−1^ for CH_2_ wagging at C-6, 1162 cm^−1^ for C–O–C anti symmetric bridge stretching vibrations, 1108 cm^−1^ for ring asymmetric stretching vibrations, and 1055 cm^−1^ for C–O–C and C–O–H stretching vibrations of the sugar ring^24^. 1031 cm^−1^ for CO stretching vibrations and 986 cm^−1^ for CO valence vibrations at C-6. 670 cm^−1^ and 618 cm^−1^ for OH out-of phase bending. These peaks suggested that BC produced from industrial waste liquid were pure cellulose. Hence the FTIR data reported that the culture medium shows little influence on the functional groups of BC samples. In addition, BC is a mixture of I_α_ and I_β_ of crystalline allomorphs^25^. The peaks derived from the I_β_ allomorph appear at 710 cm^−1^. Therefore the synthesized cellulose contained both allomorphs.

**Figure 4:**
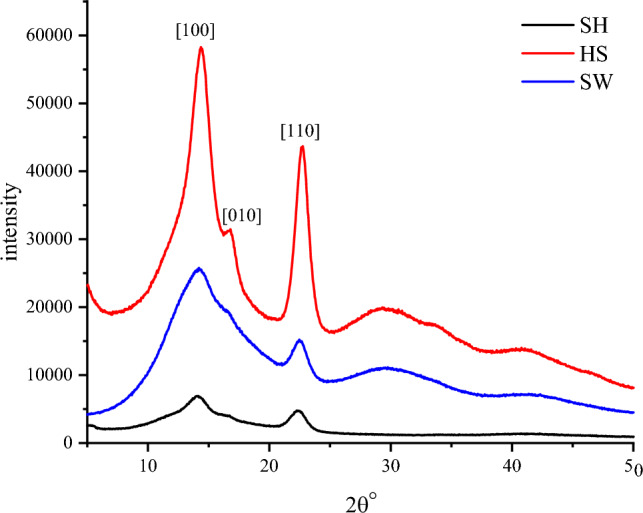
XRD patterns of BC produced from SH medium, SW medium, standard HS medium.

The morphological changes of BC are related to the changes in microstructures such as crystallinity. In order to compare the microstructural changes of BC produced from different medium, X-ray diffraction was used. The XRD patterns obtained from the BC samples are showen in Fig. 4. The obtained X-ray patterns show BC samples with the same chemical structure but with different CrI and CrS (Table 1). The characteristic peaks that appeared at 14.2° and 22.5° stood for the crystal plane [100], and [110], respectively^26^. However, the peak intensities of BC produced from SH medium and SW medium at 14.2° and 22.5° are less than those from standard HS medium, indicating that the BC synthesized using SH and SW as culture medium have much more amorphous component and less crystal component. The intensity of the [100] reflection is larger than that of the [010] one when the film is parallel to the X-ray beam and the effect is reversed in the perpendicular orientation. This reveals a strong uniplanarity that is due to the fact that the cellulose ribbons are preferentially oriented parallel to the film surface during drying. The CrI values of BC produced from standard HS medium (66.3%) were slightly higher than that of BC produced from SH medium (61.8%). The CrI of BC was reduced (46.2%) when grown in soy whey. The CrS was calculated using Scherrer's equation as 4.19 nm, 3.72 nm and 3.48 nm, respectively (Table 1). The CrS of BC produced from SH medium was smaller than that of others. The crystal size is closely related to the width of cellulose fibers^27^.

### Thermal behavior of BC

**Figure 5:**
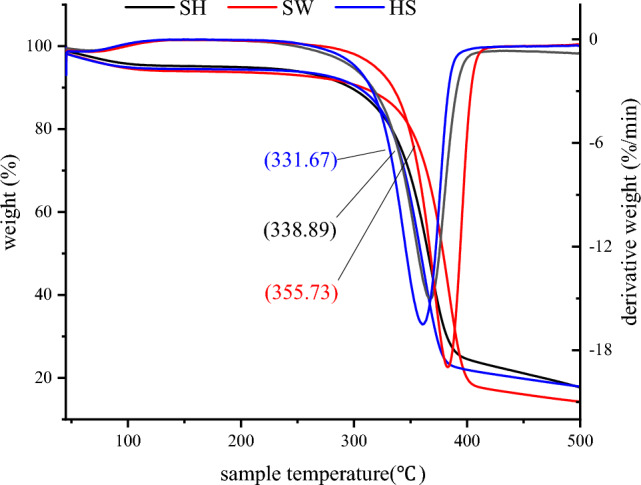
TGA of BC produced from SH medium, SW medium and standard HS medium.

To analyze the thermal stability and behavior of BC, TGA measurements were carried out on the freeze-dried BC. Figure 5 shows the TGA of BC produced from SH medium, Standard HS medium, SW medium. As shown by TGA thermograms, similar trends were observed of different BC samples, BC was recorded two distinct stages of thermal degradation, The first thermal degradation stage occurs at 90 °C to 100 °C, which is the loss of moisture content absorbed on the surface and the interlayer-coordinated water molecules^28^. The second thermal degradation stage occurs at 300 °C to 400 °C, which is the thermal degradation and cracking of BC skeleton^29^. Eventually BC was broken down into H_2_O, CO_2_, CO and volatiles. TGA results showed the highly thermal stability for BC which produced from SW medium than other BC which produced from standard HS medium and SH medium. The thermal degradation rate temperature of BC produced from SH medium, SW medium and standard HS medium were 338.89 °C, 355.73 °C. and 331.67 °C, respectively. During this phase, BC samples lost 91.4%, 90.9% and 93.5% of their original weight, respectively, and thus indicated maximum weight loss. TGA data highlights the superior thermal stability of BC produced from SH medium and SW medium.

## Conclusion

On the basis of the results, it can concluded that soybean by-product are a promising substrate for obtaining BC. These low-cost by-products can significantly contribute to the reduction of production costs. We could not only overcome the BC production cost, but also decrese the waste resources and environmental pollution. The instrumental analysis emphasized that the soybean whey and soybean hydrolysate medium can be used for fermentation production of BC. BC samples form a porous three-dimensional network structure observed by SEM and AFM. FTIR spectra showed that BC samples have the similar chemical composition. XRD study show that BC samples have different CrI and CrS. TGA data highlights the superior thermal stability of BC samples. In conclusion, food industrial byproducts soybean whey and soybean hydrolysate can be used as cost-effective medium to produce BC for large-scale industrial production as well as for the determination of their potential for application in industries concerning food, cosmetics and medicine.

### Supplementary Information

Supplementary Information.

## Data Availability

All data generated or analysed during this study are included in this published article and its supplementary information files.
