# Supplementary material for: Isolation and characterization of bacterial cellulose produced from soybean whey and soybean hydrolyzate
Source: Sci Rep. 2023 Sep 25;13:16024. doi: 10.1038/s41598-023-42304-w (PMC10520036; doi:10.1038/s41598-023-42304-w)
Supplement: Supplementary file 1 — Supplementary Information. [file 41598_2023_42304_MOESM1_ESM.doc]

Kombucha is a kind of material beneficial to human body after fermentation with sugar, tea, water and bacteria. According to analysis, its bacteria is a symbiosis of yeast, lactic acid bacteria and acetic acid bacteria. Kombucha has been spread and applied in China for more than 150 years. In recent years, the practice of Kombucha in domestic and foreign medical circles has shown that Kombucha can treat a variety of chronic diseases, such as hypertension, Arteriosclerosis, coronary heart disease, diabetes, constipation, hemorrhoids, obesity, alopecia areata, white hair, cataract, rheumatoid arthritis, gastritis, dysentery, anemia, Riboflavin deficiency, etc. *kombucha* was purchased from the China Center of Industrial Culture Collection (Beijing, China). DongNong No. 42 (DN42) soybean samples were purchased from the Northeast Agricultural University Soybean Research Institute (Harbin, Heilongjiang Province, China). Puffed soybeans were provided by Jiu San Group Tieling Soybean Technology Co., Ltd. (Tieling City, Liaoning Province, China).
